# A recombinant fungal compound induces anti-proliferative and pro-apoptotic effects on colon cancer cells

**DOI:** 10.18632/oncotarget.15859

**Published:** 2017-03-02

**Authors:** Lili Nimri, Orly Spivak, Dana Tal, Dominik Schälling, Irena Peri, Lutz Graeve, Tomer M. Salame, Oded Yarden, Yitzhak Hadar, Betty Schwartz

**Affiliations:** ^1^ The Robert H. Smith Faculty of Agriculture, Food and Environment, Institute of Biochemistry, Food Science and Nutrition, The Hebrew University of Jerusalem, Rehovot 76100, Israel; ^2^ Faculty of Natural Sciences, Institute of Biological Chemistry and Nutrition, University of Hohenheim, Stuttgart 70599, Germany; ^3^ The Robert H. Smith Faculty of Agriculture, Food and Environment, Department of Plant Pathology and Microbiology, Rehovot 76100, Israel

**Keywords:** recombinant ostreolysin, colon cancer, pro-apoptotic, fungal, microtubule

## Abstract

Finding intracellular pathways and molecules that can prevent the proliferation of colon cancer cells can provide significant bases for developing treatments for this disease. Ostreolysin (Oly) is a protein found in the mushroom *Pleurotus ostreatus*, and we have produced a recombinant version of this protein (rOly).

We measured the viability of several colon cancer cells treated with rOly. Xenografts and syngeneic colon cancer cells were injected into *in vivo* mouse models, which were then treated with this recombinant protein.

rOly treatment induced a significant reduction in viability of human and mouse colon cancer cells. In contrast, there was no reduction in the viability of normal epithelial cells from the small intestine. In the search for cellular targets of rOly, we showed that it enhances the anti-proliferative activity of drugs targeting cellular tubulin. This was accompanied by a reduction in the weight and volume of tumours in mice injected with rOly as compared to their respective control mice in two *in vivo* models.

Our results advance the functional understanding of rOly as a potential anti-cancer treatment associated with pro-apoptotic activities preferentially targeting colon cancer cells.

## INTRODUCTION

Colon cancer is the third most commonly diagnosed cancer and the fourth leading cause of cancer-related death globally [1]. Under physiological conditions, unwanted cells die by apoptosis and are quickly removed by phagocytes [2]. Apoptotic mechanisms are crucial in tumorigenesis and resistance to anti-cancer drugs. In cancer cells and tumours, recovery of the ability to conduct apoptosis or to induce alternative death pathways (such as necrosis) is very important to their survival. Functional studies over the last two decades have established that programmed cell death by apoptosis serves as a natural barrier to cancer development [3, 4]. Due to the complex physiology of cancer cells, it is extremely hard to find appropriate treatments for colon cancer. This situation has led to the urgent need to identify additional novel molecules and mechanisms involved in this pathogenesis. Such novel molecules could provide additional potential therapeutic options to inhibit carcinogenesis. Tubulin-inhibiting agents are among the common types of anti-cancerous drugs. They interfere with tubulin function during mitosis, causing the cell to eventually undergo apoptosis. There are many destabilizing agents and microtubule-stabilizing ones [5]. Nevertheless, these anti-tubulin agents have many side effects, some of which considerably affect the quality of life of the users, while others limit their efficiency. The most significant problem of the standard anti-tubulin drugs is resistance to treatments [5–7].

Here, we report on the putative anti-cancer properties of ostreolysin (Oly). Oly is a protein that belongs to the aegerolysin family of small acidic proteins found in the mushroom *Pleurotus ostreatus (P. ostreatus)*, an edible white rot ligninolytic basidiomycete fungus [8, 9]. It is a 15-kDa protein, expressed during the formation of *P. ostreatus* fruiting bodies [10, 11]. This protein specifically interacts with cholesterol-enriched raft-like membrane domains (lipid rafts) [12, 13]. Cancer cell membranes are enriched with lipid rafts [14], which can be used to test the potential of Oly as an effector of cancer cell apoptosis. As the natural expression levels of this protein in the fungus are very low, and it has been found to be haemolytic in its dimer form [15, 16], we recently prepared a novel recombinant version, expressed in *Escherichia coli* (Supplementary Methods and Supplementary Figures 1–3). In contrast to the haemolytic activity of natural Oly on bovine, sheep, human and rat erythrocytes [12, 13], rOly had no such effect in mice. Because of the apparent safety of this compound in preliminary *in vivo* experiments conducted in our laboratory, we used it to test cancer-treatment effectiveness in *in vivo* models. Moreover, we tested its effect *in vitro* on the viability of several colon cancer cell lines of mouse and human origin. We suggest that rOly can be further studied as an effective novel pro-apoptotic specific anti-cancer drug.

## RESULTS

### rOly penetrates the cell membrane and enters the cytosol in HCT116 cells

**Figure 1:**
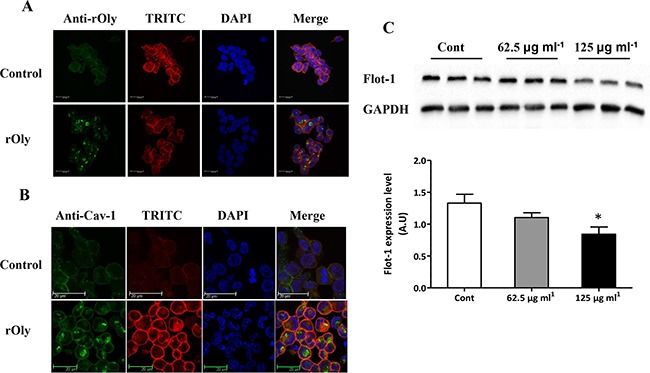
Recombinant ostreolysin (rOly) penetrates the cell membrane and enters the cytosol of HCT116 cells **A, B**. Representative immunofluorescence of HCT116 cells treated for 8 h with control medium (top panel) or with medium containing 125 μg ml^−1^ rOly (bottom panel). (A) Presence of rOly inside the cells, recognized by anti-rOly antibody. Nuclei were counterstained with DAPI and filamentous actins were counterstained with TRITC. Scale bars = 20 μm. This is one representative section out of three independent experiments. (B) Clustering of caveolin-1 (Cav-1) on the membrane, recognized by anti-Cav-1 antibody. Scale bars = 20 μm. One representative section out of three independent experiments is presented. **C**. Untreated cells (Cont) or cells treated for 8 h with rOly (62.5 or 125 μg ml^−1^) were analysed by western blot, and densitometry analysis of the western blot data was performed. Values are means ± SEM, n = 9–15. *P < 0.05 vs. Cont (one way ANOVA–Dunnett's test). The results are presented as means of four independent experiments.

Cells treated for 8 h with 125 μg ml^−1^ rOly presented a clear total cell distribution of this recombinant protein. In addition to membrane clustering, cross-sectional images of rOly-treated cells demonstrated that it penetrates the cell membrane and enters the cytosol (Figure 1A).

Cholesterol–sphingolipid-rich domains typically contain caveolins [17] and can be recognized by rOly. HCT116 colon cancer cells treated for 8 h with 125 μg ml^−1^ rOly presented more Caveolin-1 (Cav-1) -rich domains than control cells. However, the labelling pattern of the two proteins indicated that they are not co-localized (Figure 1B).

In addition, we investigated the membrane distribution of the lipid raft-associated protein flotillin-1 (Flot-1) in cells treated with rOly. HCT116 colon cancer cells treated for 8 h with 125 μg ml^−1^ rOly exhibited less Flot-1-rich domains than the untreated cells (Figure 1C).

### Effect of rOly treatment on colon cancer cell viability *in vitro*

**Figure 2:**
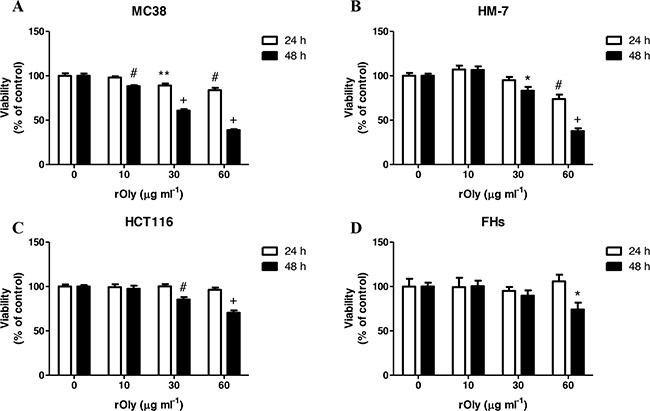
Recombinant ostreolysin (rOly) reduces colon cancer cell viability but not normal cell viability Cells were grown overnight in DMEM and then treated with different concentrations of rOly for 24 and 48 h. Cell viability was estimated by MTT assay. *P < 0.05, **P < 0.01, ^#^*P* < 0.001, ^+^*P* < 0.0001 as compared to control; n = 8–10 (Student's *t*-test). The control was set to 100%. Data are presented as means ± SEM of two independent experiments.

Exposure of MC38 cells to rOly produced a significant decrease in their viability at almost all of the rOly concentrations tested for 24 and 48 h. The highest reduction in cell viability was observed after 48 h of treatment at 30 and 60 μg ml^−1^ (40% and 61%, respectively) (Figure 2A). A similar trend was observed in HM-7 human colon cancer cells, where the most significant reduction in viability was seen at 60 μg ml^−1^ rOly for both treatment periods, with a 27% reduction after 24 h and 62% reduction after 48 h (Figure 2B). In the case of HCT116 human colon cancer cells, there was a significant reduction in viability, by 30% after 48 h of incubation with 60 μg ml^−1^ rOly. No significant change was seen after 24 h (Figure 2C). Interestingly, the viability of rOly-treated FHs normal cells was similar to control cells after 24 and 48 h of treatment with 10 and 30 μg ml^−1^ rOly. A borderline effect was measured after treating the cells for 48 h with the highest concentration of rOly (60 μg ml^−1^). However, this reduction was significantly smaller than that effected by rOly in the other cell lines at similar time periods and concentrations (Figure 2D).

### rOly treatment of HCT116 cells induces apoptosis

**Figure 3:**
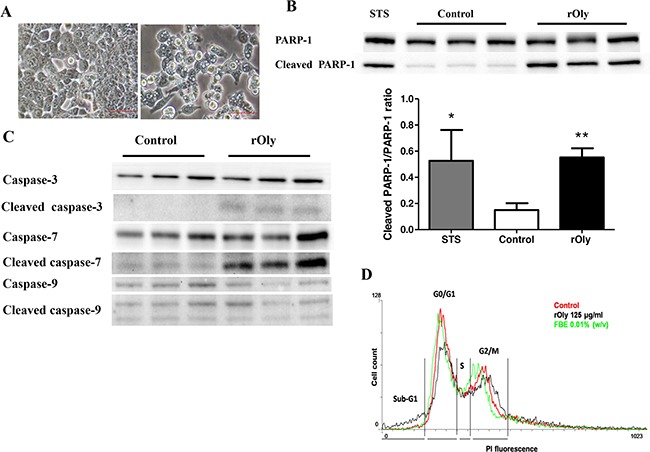
Recombinant ostreolysin (rOly) induces apoptosis in HCT116 cells **A**. Morphological changes after 8 h of rOly stimulation. HCT116 cells in the absence of rOly (left) compared to HCT116 cells treated with 125 μg ml^−1^ rOly (right). Scale bars = 50 μm. **B**. Cells were treated, or not (Control), with 125 μg ml^−1^ rOly for 8 h. PARP-1 (top panel) and cleaved PARP-1 (bottom panel) proteins were detected by western blot, and densitometry analysis of the western blot data was performed. Cells treated with 1 μM staurosporine (STS) were used as positive controls. Values are means ± SEM (STS n = 3, Control and rOly n = 8). *P < 0.05 and **P < 0.01 vs. control (one-way ANOVA–Dunnett's test). The results are presented as means of three independent experiments. **C**. Cells were treated, or not (Control), with 125 μg ml^−1^ rOly for 8 h. Expression levels of caspase-3, cleaved caspase-3, caspase-7, cleaved caspase-7, caspase-9 and cleaved caspase-9 proteins were detected by western blot. This is one representative experiment out of three independent experiments. **D**. Cells were treated with 125 μg ml^−1^ rOly or 0.01% fruiting body extract (FBE) or were untreated (Control) for 8 h. Control (Red), FBE (Green), rOly (Black). Results are representative of one out of two independent experiments performed in triplicate. Data were obtained from 15,000 HCT116 cells.

rOly treatment induced noticeable morphological changes in HCT116 cells compared to controls (Figure 3A). The cells lost their elongated shape and concomitantly accumulated intracellular droplet-like structures of different sizes that filled the cytoplasm. To determine whether these structures reflect a pro-apoptotic effect conferred by rOly, we analysed the presence of several apoptotic markers in the treated cells. Western blot examination of apoptotic marker proteins revealed that 125 μg ml^−1^ rOly induces significant cleavage of poly (ADP-ribose) polymerase-1 (PARP-1), with significantly higher levels of the cleaved protein (*P* < 0.05) seen in the treated cells (Figure 3B). Examination of full-length and cleaved caspase proteins also indicated that apoptosis had occurred in the rOly-treated cells; even though caspase-9 full length and cleaved protein levels remained unchanged, the cleaved protein levels of the executioner caspases (3, 7) were higher in rOly-treated cells than in controls (Figure 3C).

To quantify cell distribution during apoptosis, HCT116 cells treated with 125 μg ml^−1^ rOly or 0.01% fruiting body extract (FBE) were analysed by flow cytometry. At the sub-G1 (apoptosis) peak, the differences between untreated and rOly-treated cells were significant (*P* < 0.01; Table 1). After staining with a quantitative DNA-binding dye, cells that have lost DNA via apoptosis will take up less stain and will appear as a sub-G1 peak to the left of the G0/G1 peak (Figure 3D).

**Table 1:** Cell-cycle analyses of HCT116 cells following different treatments

	Control	rOly (125 μg ml^−1^)	FBE 0.01% (w/v)
Sub-G1	1.2 ± 0.1	7.3 ± 0.5	2.0 ± 0.3
G0/G1	42.4 ± 1.7	33.8 ± 1.1	46.0 ± 2.3
S	13.1 ± 0.8	11.4 ± 0.7	13.0 ± 1.1
G2/M	27.9 ± 0.8	27.5 ± 1.1	25.9 ± 0.4

Cell cycle of untreated HCT116 cells or cells treated with 125 μg ml-1 rOly or 0.01% (w/v) of fruiting body extract (FBE) was analyzed using WinMDI 2.9 software. All cell phases are represented as relative percent of all cells. Cells not identified in cell phases were also counted. Data shown are the mean ± SE of two independent experiments performed in triplicate. Data were obtained from 15,000 HCT116 cells.

### rOly binds to β-tubulin and affects HCT116 cell viability

**Figure 4:**
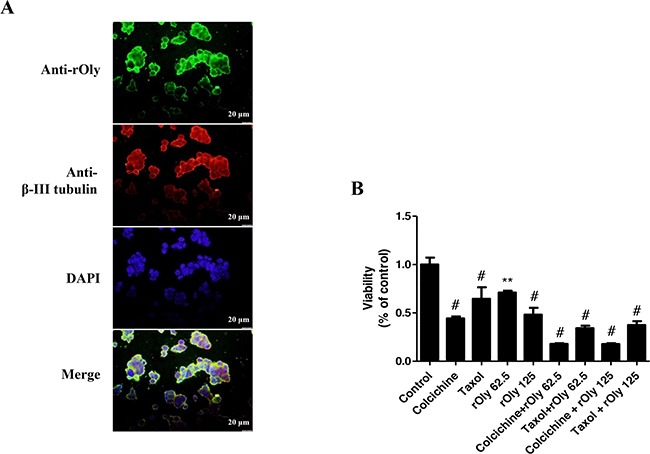
Recombinant ostreolysin (rOly) and tubulin-inhibiting agents decrease HCT116 cell viability **A**. Immunofluorescence of β-III tubulin and rOly in HCT116 cells. Cells were treated for 90 min with 125 μg ml^−1^ rOly and labelled with specific anti-rOly (green) and anti-β-III tubulin (red) antibodies. Nuclei were counterstained with DAPI. Scale bars = 20 μm. This is one representative section out of three independent experiments. **B**. HCT116 cells were grown overnight in DMEM and then subjected to different treatments as described in Materials and Methods. Cell viability was estimated by MTT assay. ^#^*P* < 0.001, **P < 0.01 as compared to control, n = 6 (one-way ANOVA–Dunnett's test). Data are presented as means ± SEM of one independent experiment out of three.

We investigated the putative binding partners of rOly. We observed clear co-labelling of tubulin and rOly in HCT116 cells treated with rOly, indicating that these proteins co-localize within the cell following treatment (Figure 4A).

Our next goal was to determine whether rOly affects the viability of cancer cells through mechanisms similar to those of tubulin-inhibiting agents, i.e., apoptosis induction through similar pathways. To this end, we chose two known tubulin inhibitors: a destabilizing agent (colchicine) and a microtubule-stabilizing agent (taxol). Results showed that 10 mM colchicine and 1 μM taxol significantly reduce cell viability, by 66% and 36%, respectively; 125 mg ml^−1^ rOly was sufficient to reduce the viability of the cells by 52%, while 62.5 mg ml^−1^ rOly had a lesser (28%), albeit significant, effect. rOly's effect on the cells’ viability was significantly enhanced at both concentrations of rOly following anti-tubulin treatment. Consequently, the cells’ viability decreased as a result of each treatment. However, a higher reduction in cell viability, which reached more than 80%, was measured when cells were treated with both colchicine and rOly at both concentrations (62.5 mg ml^−1^ and 125 mg ml^−1^) (Figure 4B).

### rOly inhibits tumour growth in two *in vivo* colon cancer models

To assess the ability of rOly to induce anti-proliferation *in vivo*, we used two models: *nu/nu* athymic mouse xenografts and C57BL/6 mice. We used the immunodeficient *nu/nu* athymic mice to ensure that the effect exerted by rOly in C57BL/6 mice is not a result of non-specific stimulation of their immune system.

**Figure 5:**
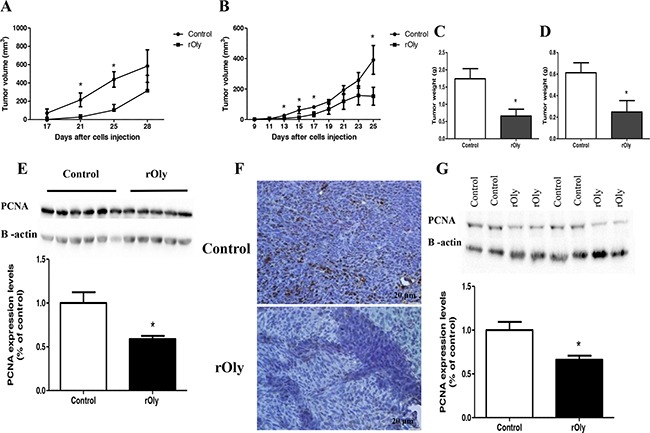
Recombinant ostreolysin (rOly) delays tumour growth and reduces tumour weight in mice MC38 mouse colon cancer cells (2 × 10^5^) and HCT116 human colon cancer cells (1×10^6^) were subcutaneously injected into C57BL/6 mouse's right rear or into the left hip of athymic nude mice respectively. C57BL/6 mice were sacrificed on day 28 and athymic nude mice on day 25. Tumour tissues were excised and weighed. Tumour volume and weight of C57BL/6 mice **A, C**. and of *nu/nu* athymic mice **B, D**. were measured. **A, C**. n = 5–6 for control and rOly groups. **B, D**. n = 8 for the control group and n = 11–12 for the rOly group. *P < 0.05 (Student's *t*-test). **E**. Tumour tissues from C57BL/6 mice were analysed by western blot and densitometry analysis (n = 6 for control group, n = 5 for rOly group). *P < 0.05 (Student's *t*-test). **F**. Immunohistochemistry was performed on tumours of C57BL/6 mice from each group using anti-PCNA antibody. PCNA protein was localized to the cell nucleus of the tumours. Nuclei were counterstained with haematoxylin. Scale bars = 20 μm. This is one representative section from one mouse out of 5–6 different mice. **G**. Tumour tissues from *nu/nu* athymic mice were analysed by western blot and densitometry analysis (n = 8 for control group, n = 5 for rOly group). *P < 0.05 (Student's *t*-test).

The tumour volume in mice of both models was significantly lower in the mice injected with rOly compared to the control group (Figure 5A and 5B; *P* < 0.05). It is important to mention that all control mice, from both genotypes, eventually developed tumours. In contrast, only 3 of 6 and 5 of 12 rOly-injected mice developed tumours in the C57BL/6 and *nu/nu* athymic models, respectively. After sacrificing the mice, the tumours were weighed. The tumours of the rOly group weighed significantly (*P* < 0.05) less than those of the control group in both mouse models (Figure 5C and 5D).

Immunostaining and western blot analysis with an anti-proliferating cell nuclear antigen (PCNA) antibody in the C57BL/6 mouse model (Figure 5E and 5F) revealed low PCNA expression and staining in the tumour sections from the rOly-treated group as compared to controls. The same trend was seen in *nu/nu* athymic mouse tumours (Figure 5G). These results demonstrated reduced proliferation of cancer cells in the tumours of mice treated with rOly.

## DISCUSSION

The edible mushroom *Pleurotus* is considered a health food with very high nutritional value [18]. In addition, glucans extracted from *Pleurotus* species have been shown to exhibit a wide variety of medicinal properties [19]. A low-molecular-weight α-glucan extracted from *P. ostreatus* demonstrated anti-proliferative properties against HT-29 colon cancer cells by inducing programmed cell death [20]. Here, we demonstrated that rOly, a 15-kDa protein originally expressed by *P. ostreatus*, promotes apoptotic death of the colon cancer cell lines HCT116, HM-7 and MC38 and inhibits growth of tumours in C57BL/6 and nude mice.

Previous studies have demonstrated that Oly extracted from *P. ostreatus* induces perforation of membranes that contain high levels of cholesterol- and sphingolipid-rich lipid rafts [12, 21]. This was further supported by showing that the extracted protein interacts exclusively with membranes that are rich in cholesterol and sphingomyelin [22]. However, rOly does not permeabilize these membranes [15], but as shown here, it does induce significant morphological changes in the shape of these cells, probably mirroring the apoptotic process. Furthermore, it has been shown that perforation activity of the native 15-kDa Oly is due to incomplete separation from the native 56-kDa protein pleurotolysin B [15]. Native Oly can be cytotoxic to various cells because it allows permeabilization of membranes that are rich in cholesterol and sphingomyelin by dimerization with pleurotolysin B. Pleurotolysin B can insert itself into the membrane lipid barrier through its restructuring of particular helices into hairpin β-strands, promoting pore formation [15]. Our cell-cycle analyses are in line with Ota *et al*'s (2013) observations since we demonstrated that FBE prepared from *P. ostreatus* and containing native Oly inhibits cell proliferation but does not induce cell apoptosis. Collectively, the results show that rOly is unable to perforate cells by itself but is internalized into the cell by an unknown mechanism. We report herein on specific changes that take place in colon cancer cells in response to rOly treatment: we found that shortly after binding to the cell membrane, rOly is internalized. A similar phenomenon has been previously reported by Skocaj *et al* (2014) [23]. We surmise that after its internalization, rOly binds cytosolic proteins; we clearly demonstrated, by co-localization analyses, that one of these proteins is β-III tubulin, a known target for chemotherapeutic drugs. We assume that the interaction of rOly with tubulin affects microtubule dynamics, eventually causing the cells to undergo apoptosis. Once rOly is internalized in the cell, it binds to β-III tubulin, and we assume that rOly directly promotes cells to undergo apoptosis in a tubulin binding-dependent mechanism of action, as has also been seen for other anti-cancer agents and chemotherapeutic drugs [24]. The series of events from tubulin interaction to activation of the apoptotic pathway remains to be further clarified.

We showed that rOly interacts with certain proteins of the membrane domains known as lipid rafts. One such protein is Flot-1, which has been reported in various studies as being pro-carcinogenic [25, 26]. We assume that this interaction leads to programmed cell death (apoptosis). rOly might be a putative regulator of Flot-1 and eventually affect pathways involving this cellular protein, such as epidermal growth factor (EGF) receptor signalling.

Polymerization of caveolins has been shown to lead to clustering and invagination of existing cholesterol–sphingolipid-rich domains in the cell plasma membrane [17]. We clearly demonstrated that rOly penetrates the cell membrane and enters into the cytosol. We assume that Cav-1 regulates cell penetration of rOly and as a result, rOly reduces the level of Flot-1 in the lipid raft, leading to less EGF anchoring to the cell membrane and thereby promoting apoptosis.

We found that rOly is able to promote programmed cell death of colon cancer cells in a dose-dependent manner. However, this apoptotic effect was less significant in cancer cells with medium malignant ability (HCT116) and not significant in a normal intestinal cell line (FHs). To our knowledge, this is the first study showing specific apoptotic ability of rOly on colon cancer cells, a process that is not induced in normal intestinal cells.

Changes in mitochondrial membrane permeability and the subsequent release of pro-apoptotic factors are involved in various aspects of apoptosis [27]. A universal hallmark of apoptosis is the proteolytic cleavage and inactivation PARP-1 in the execution phase of cell death. PARP-1 prevents the cell from undergoing apoptosis when the cell can repair DNA damage. Cleavage of PARP-1 is an indication that the cell is unable to cope with saturating DNA injury [28]. PARP-1 itself can be inactivated by caspase cleavage, and it is a well-known substrate of caspase-3 [29]. Our results show that rOly promotes the cleavage of caspases 3, 9 and 7, promoting the inactivation and cleavage of PARP-1 and indicating induction of apoptosis. Thus, we cannot conclude by which apoptotic program rOly triggers cell death. Nevertheless, activation of the effector caspase-3 provides evidence that rOly is capable of stimulating apoptosis of HCT116 cells.

Our main reason for using of two different kinds of anti-tubulin agents was to examine the mechanism governing rOly induction of apoptosis after binding to the cell's microtubules. We wanted to clarify whether inhibition of cell microtubule dynamics is involved in apoptosis induced by rOly. Therefore, cells were pre-treated with either taxol or colchicine, to disturb tubulin polymerization or dynamics, and only afterwards was 62.5 or 125 mg ml^−1^ rOly added. Decreased viability was observed when using rOly with either of the two agents. Therefore, we concluded that rOly is able to induce apoptosis in the presence of either free tubulin subunits or altered microtubule dynamics; its effect is on specific tubulin sequences and does not depend on their ordered structure.

Finally, we conducted *in vivo* experiments to test the effect of rOly on tumour growth in mice. Tumour growth rate was significantly down-regulated in mice treated with rOly, and there was a significant reduction in tumour weight in the rOly versus control group. Moreover, we demonstrated by both western blot and immunohistochemical analysis that expression level of PCNA in the tumours of rOly-treated mice is significantly lower than in tumours originating from mice belonging to the control group. In addition, significantly less blood cells were detected in the tumours of rOly-treated mice as compared to control mice (data not shown). These findings could implicate a weakened inflammatory process in the tumour tissue, and/or angiogenesis in the tumours from mice treated with rOly. We conclude that rOly directly modulates apoptotic signal-transduction pathways and may concomitantly affect inflammatory processes in the tumour tissue.

Our study demonstrates rOly's potential as an anti-cancer agent, capable of inhibiting tumour growth in nude mice, induce apoptosis in colon cancer cell lines and bind *in vitro* to several isoforms of tubulin, including β-III tubulin. Therefore, it might be considered as a novel pharmaceutical agent to combat cancer. rOly has advantages over some of the familiar anti-cancer drugs used in the clinic today: its high solubility in water and low molecular weight increase its bioavailability in comparison to other drugs; its high affinity to cholesterol- and sphingomyelin-rich membranes might reduce the resistance effect of efflux pumps and contribute to its specificity towards tumour cells.

## MATERIALS AND METHODS

### Materials

All chemicals and biochemicals were from Sigma Chemical Co. (St. Louis, MO, USA), unless otherwise specified. rOly was produced as described in Supplementary Methods and Supplementary Figures 1–3.

### Cell culture

MC38 is a colon adenocarcinoma cell line of C57BL/6 origin [30]. MC38 cells were maintained as previously described [31]. The human colon cancer cell lines HCT116 and HM-7 were maintained as previously described [32]. FHs 74 Int cells, which are normal epithelial cells from the small intestine, were maintained in Hybri-care medium supplemented with 10% (v/v) fetal bovine serum (FBS) and 0.2% (v/v) penicillin–streptomycin–nystatin. All cells were kept at 37 °C with 5% CO_2_.

### Immunofluorescence

HCT116 cells were seeded and allowed to adhere overnight. The cells were treated with Dulbecco's modified Eagle's medium (DMEM) with or without 125 μg ml^−1^ or 15 μg ml^−1^ rOly. Cells were fixed in 3.7% (v/v) formaldehyde in phosphate-buffered saline (PBS) with 0.5% (v/v) Triton X-100, blocked in PBS containing 5% (v/v) donkey serum (Jackson, New Baltimore, PA, USA), and incubated with rabbit polyclonal anti-caveolin-1 (anti-Cav-1) (Santa Cruz Biotechnology, Santa Cruz, CA, USA) with mouse monoclonal anti-β-III tubulin (Cell Signaling Technology, Danvers, MA, USA) or anti-rOly specially prepared for our group by Adar Biotech Ltd. (Rehovot, Israel). Cells were incubated with Alexa Fluor® 488 (ab150077) goat anti-rabbit IgG secondary antibody (Abcam, Cambridge, UK) and phalloidin- Tetramethylrhodamine (Phalloidin-TRITC). Nuclei were stained with 1 mg ml^−1^ 4′6-diamidino-2-phenylindole (DAPI). Stained cells were examined under an inverted light microscope (Nikon Eclipse E400) at 40X magnification.

### Cell-cycle analysis

HCT116 cells were plated and allowed to adhere overnight. Cells were treated, or not, with 125 μg ml^−1^ rOly, and *P. ostreatus* fruiting body extract (FBE) was added at a concentration of 0.01% (w/v). *P. ostreatus* FBE was first examined and used in our laboratory as a reference for the rOly haemolytic assay since it includes native Oly. DNA content was measured by excitation of propidium iodide (PI) at 488 nm and measuring the emission at 575 nm (FL2) using a flow cytometer (BD FACScalibur BD Biosciences, San Jose, CA). Analysis was performed with WinMDI 2.9 software.

### Animals and xenograft cancer model

Animal care and experimental procedures were in accordance with the guidelines of the accredited animal ethics committee of the Hebrew University of Jerusalem.

The experiment using the C57BL/6 model was essentially conducted as previously described [31] with small modifications: 11-week-old C57BL/6 male mice (n = 12) weighing 20–26 g were purchased from Harlan (Jerusalem, Israel). Mice were randomly divided into a control group and a rOly group, 6 mice per group. MC38 mouse colon cancer cells (2 × 10^5^) suspended in 0.1 ml DMEM were subcutaneously injected into C57BL/6 mouse's right rear flank. Three days after injection, the rOly and control mouse groups were injected twice a week via the peritoneal cavity with a fixed concentration of rOly (1 μg g^−1^ body weight [BW]) or saline, respectively. One week later, mice were injected every second day with rOly (1 μg g^−1^ BW) or saline for a total experimental period of 28 days. Seventeen days after cell injection, tumour volume was measured twice a week until the mice were sacrificed on day 28. Tumour tissues were excised and weighed, and tumour aliquots were transferred into 4% paraformaldehyde for immunohistology or into lysis buffer for western blot analysis.

For the athymic *nu/nu* model, 6- to 7-week-old CD-1 *nu/nu* mice, weighing 25–30 g, were randomly divided (12 mice in the rOly group, 9 mice in the control group) and housed in appropriate specific pathogen-free (SPF) cages. HCT116 human colon cancer cells (1×10^6^) suspended in 0.1 ml saline were injected subcutaneously into the left hip of athymic nude mice. Starting 2 days after injection, mice were treated every other day with intraperitoneal injections of rOly (1.5 μg g^−1^ BW) or saline for a total experimental period of 25 days. The tumours were measured every second day as they appeared, and the corresponding volumes were estimated until the mice were sacrificed on day 25. Tumour tissues were excised and weighed, and samples were transferred into lysis buffer for western blot analysis.

### Immunohistochemistry

Tissue (5-μm thick sections) was processed, incubated with the primary antibody anti-proliferating cell nuclear antigen (PCNA) (Santa Cruz Biotechnology, Dallas, TX, USA) and examined with an inverted light microscope (Nikon Eclipse E400) at 40X magnification as described previously [33].

### Western blotting

Tissues or cells were lysed and 30–60 μg protein were subjected to SDS-PAGE, transferred to nitrocellulose membranes (Whatman, Schleicher & Schuell, Dassel, Germany) and blocked in 5% (w/v) dry nonfat milk (Difco, Sparks, MD, USA) as described previously [31]. Membranes were incubated with primary rabbit polyclonal antibodies: anti-Flotillin-1, anti-PCNA (Santa Cruz Biotechnology), anti-GAPDH (Abcam), anti- poly (ADP-ribose) polymerase-1 (PARP-1), anti-cleaved caspase-9, anti-caspase-9 (Cell Signaling Technology) and anti-β-actin (Sigma–Aldrich). Primary rabbit monoclonal antibodies included: anti-cleaved PARP-1, anti-cleaved caspase-3, anti-caspase-3, anti-caspase-7, and anti-cleaved caspase-7 (Cell Signaling Technology). Secondary antibodies were obtained from Jackson ImmunoResearch (West Grove, PA, USA). Proteins were visualized using an enhanced chemiluminescence kit. Densitometry was assessed using the Gelpro32 analyzer software.

### Cell viability assay

Human or mouse colon cancer cells with different malignant abilities (HM-7, HCT116, and MC38) and non-malignant small intestine cells (FHs) were plated on multiple 96-well plates in DMEM for 24 h and treated, or not, with rOly at a concentration of: 10 μg ml^−1^, 30 μg ml^−1^ and 60 μg ml^−1^ for an additional 24 and 48 h. In studies involving tubulin-inhibiting agents, HCT116 cells were treated, or not, with rOly (62.5 or 125 μg ml^−1^) for 24 h, with 10 mM colchicine or 1 mM taxol for 4 h, followed by addition of fresh medium or medium supplemented with rOly (62.5 or 125 μg ml^−1^) for an additional 20 h. Cells were then incubated with 3-(4,5- dimethylthiazol-2-yl)-2,5 diphenyltetrazoliumbromide (MTT, 0.5 mg ml^−1^) for 1 h followed by incubation with DMSO for 20 min. Formation of the coloured formazan dye was assessed colorimetrically at 550 nm in an ELX 808 Ultra microplate reader (Bio-Tek Instruments, London, UK) using KCJunior software (York, UK).

### Data analysis

Statistical analyses were performed by one-way ANOVA–Dunnett's test and by two-tailed Student's *t*-test. Statistical analyses were carried out using GraphPad Prism software and the *P* values are indicated. Results are presented as mean ± SEM. All figures show representative results of at least two independent experiments. Differences were considered significant at *P* < 0.05.

## SUPPLEMENTARY MATERIALS FIGURES AND TABLES



## Abbreviations

rOly: Recombinant Ostreolysin
*P. Ostreatus*: *Pleurotus ostreatus*
FBE: Fruiting body extract
EGF: epidermal growth factor
Cav-1: Caveolin-1
Flot-1: Flotillin-1
PCNA: proliferating cell nuclear antigen
PARP-1: poly (ADP-ribose) polymerase-1

## References

[1] Brenner H, Stock C, Hoffmeister M (2015). Colorectal cancer screening: the time to act is now. BMC Med.

[2] Lauber K, Bohn E, Krober SM, Xiao YJ, Blumenthal SG, Lindemann RK, Marini P, Wiedig C, Zobywalski A, Baksh S, Xu Y, Autenrieth IB, Schulze-Osthoff K (2003). Apoptotic cells induce migration of phagocytes via caspase-3-mediated release of a lipid attraction signal. Cell.

[3] Adams JM, Cory S (2007). The Bcl-2 apoptotic switch in cancer development and therapy. Oncogene.

[4] Lowe SW, Cepero E, Evan G (2004). Intrinsic tumour suppression. Nature.

[5] Perez EA (2009). Microtubule inhibitors: Differentiating tubulin-inhibiting agents based on mechanisms of action, clinical activity, and resistance. Mol Cancer Ther.

[6] Gottesman MM (2002). Mechanisms of cancer drug resistance. Annu Rev Med.

[7] Pellegrini F, Budman DR (2005). Review: tubulin function, action of antitubulin drugs, and new drug development. Cancer Invest.

[8] Cohen R, Persky L, Hadar Y (2002). Biotechnological applications and potential of wood-degrading mushrooms of the genus Pleurotus. Appl Microbiol Biotechnol.

[9] Kues U, Liu Y (2000). Fruiting body production in Basidiomycetes. Appl Microbiol Biotechnol.

[10] Berne S, Krizaj I, Pohleven F, Turk T, Macek P, Sepcic K (2002). Pleurotus and Agrocybe hemolysins, new proteins hypothetically involved in fungal fruiting. Biochim Biophys Acta.

[11] Berne S, Sepcic K, Anderluh G, Turk T, Macek P, Poklar Ulrih N (2005). Effect of pH on the pore forming activity and conformational stability of ostreolysin, a lipid raft-binding protein from the edible mushroom Pleurotus ostreatus. Biochemistry.

[12] Sepcic K, Berne S, Potrich C, Turk T, Macek P, Menestrina G (2003). Interaction of ostreolysin, a cytolytic protein from the edible mushroom Pleurotus ostreatus, with lipid membranes and modulation by lysophospholipids. Eur J Biochem.

[13] Zuzek MC, Macek P, Sepcic K, Cestnik V, Frangez R (2006). Toxic and lethal effects of ostreolysin, a cytolytic protein from edible oyster mushroom (Pleurotus ostreatus), in rodents. Toxicon.

[14] Fan JY, Carpentier JL, van Obberghen E, Grunfeld C, Gorden P, Orci L (1983). Morphological changes of the 3T3-L1 fibroblast plasma membrane upon differentiation to the adipocyte form. J Cell Sci.

[15] Ota K, Leonardi A, Mikelj M, Skocaj M, Wohlschlager T, Kunzler M, Aebi M, Narat M, Krizaj I, Anderluh G, Sepcic K, Macek P (2013). Membrane cholesterol and sphingomyelin, and ostreolysin A are obligatory for pore-formation by a MACPF/CDC-like pore-forming protein, pleurotolysin. B. Biochimie.

[16] Tomita T, Noguchi K, Mimuro H, Ukaji F, Ito K, Sugawara-Tomita N, Hashimoto Y (2004). Pleurotolysin, a novel sphingomyelin-specific two-component cytolysin from the edible mushroom Pleurotus ostreatus, assembles into a transmembrane pore complex. J Biol Chem.

[17] Parton RG, Simons K (2007). The multiple faces of caveolae. Nat Rev Mol Cell Biol.

[18] Valverde ME, Hernandez-Perez T, Paredes-Lopez O (2015). Edible mushrooms: improving human health and promoting quality life. Int J Microbiol.

[19] Lavi I, Levinson D, Peri I, Nimri L, Hadar Y, Schwartz B (2010). Orally administered glucans from the edible mushroom Pleurotus pulmonarius reduce acute inflammation in dextran sulfate sodium-induced experimental colitis. Br J Nutr.

[20] Lavi I, Friesem D, Geresh S, Hadar Y, Schwartz B (2006). An aqueous polysaccharide extract from the edible mushroom Pleurotus ostreatus induces anti-proliferative and pro-apoptotic effects on HT-29 colon cancer cells. Cancer Lett.

[21] Chowdhury HH, Rebolj K, Kreft M, Zorec R, Macek P, Sepcic K (2008). Lysophospholipids prevent binding of a cytolytic protein ostreolysin to cholesterol-enriched membrane domains. Toxicon.

[22] Sepcic K, Berne S, Rebolj K, Batista U, Plemenitas A, Sentjurc M, Macek P (2004). Ostreolysin, a pore-forming protein from the oyster mushroom, interacts specifically with membrane cholesterol-rich lipid domains. FEBS Lett.

[23] Skocaj M, Resnik N, Grundner M, Ota K, Rojko N, Hodnik V, Anderluh G, Sobota A, Macek P, Veranic P, Sepcic K (2014). Tracking cholesterol/sphingomyelin-rich membrane domains with the ostreolysin A-mCherry protein. PLoS One.

[24] Jordan MA (2002). Mechanism of action of antitumor drugs that interact with microtubules and tubulin. Curr Med Chem Anticancer Agents.

[25] Asp N, Pust S, Sandvig K (2014). Flotillin depletion affects ErbB protein levels in different human breast cancer cells. Biochim Biophys Acta.

[26] Banning A, Kurrle N, Meister M, Tikkanen R (2014). Flotillins in receptor tyrosine kinase signaling and cancer. Cells.

[27] Orrenius S (2004). Mitochondrial regulation of apoptotic cell death. Toxicol Lett.

[28] Oliver FJ, de la Rubia G, Rolli V, Ruiz-Ruiz MC, de Murcia G, Murcia JM (1998). Importance of poly(ADP-ribose) polymerase and its cleavage in apoptosis. Lesson from an uncleavable mutant. J Biol Chem.

[29] Lu X, Li C, Wang YK, Jiang K, Gai XD (2014). Sorbitol induces apoptosis of human colorectal cancer cells via p38 MAPK signal transduction. Oncol Lett.

[30] Tirapu I, Arina A, Mazzolini G, Duarte M, Alfaro C, Feijoo E, Qian C, Chen L, Prieto J, Melero I (2004). Improving efficacy of interleukin-12-transfected dendritic cells injected into murine colon cancer with anti-CD137 monoclonal antibodies and alloantigens. Int J Cancer.

[31] Nimri L, Saadi J, Peri I, Yehuda-Shnaidman E, Schwartz B (2015). Mechanisms linking obesity to altered metabolism in mice colon carcinogenesis. Oncotarget.

[32] Yehuda-Shnaidman E, Nimri L, Tarnovscki T, Kirshtein B, Rudich A, Schwartz B Secreted human adipose leptin decreases mitochondrial respiration in HCT116 colon cancer cells. PLoS One.

[33] Algamas-Dimantov A, Yehuda-Shnaidman E, Hertz R, Peri I, Bar-Tana J, Schwartz B (2014). Prevention of diabetes-promoted colorectal cancer by (n-3) polyunsaturated fatty acids and (n-3) PUFA mimetic. Oncotarget.

