# Supplementary material for: A recombinant fungal compound induces anti-proliferative and pro-apoptotic effects on colon cancer cells
Source: Oncotarget. 2017 Mar 2;8(17):28854–64. doi: 10.18632/oncotarget.15859 (PMC5438697; doi:10.18632/oncotarget.15859)
Supplement: Supplementary file 1 [file oncotarget-08-28854-s001.pdf]

A recombinant fungal compound induces anti-proliferative and pro-apoptotic effects on colon cancer cells

SUPPLEMENTARY METHODS

RNA purification, reverse transcription and cDNA cloning

Total RNA was purified from the fruiting bodies of *Pleurotus ostreatus* using the RNeasy Plant Mini Kit (Qiagen) according to the manufacturer's protocol. Up to 5 µg RNA was used for cDNA synthesis using SuperScript™ III First-Strand Synthesis System for RT-PCR (Invitrogen) according to the manufacturer's protocol. Ostreolysin (Oly) cDNA was amplified by PCR using the primers (F: 3'-ATGGCATACGCACAATGG-5'; R: 3'-TTAGT TCCCCTTCTTCAAGGTGT-5'), purified from a 1% (w/v) agarose gel and transformed into *Escherichia coli* JM109 cells (Promega) using the pGEM-T Vector System II (Promega) according to the manufacturer's protocol. The resulting plasmid was purified using QIAprep Spin Miniprep Kit (Qiagen) according to the manufacturer's protocol. This plasmid, designated pOly, was completely sequenced.

Recombinant Oly (r-Oly) expression and preparation

Oly was PCR-amplified from pOly using primers containing NcoI (at the 5' end) and BamHI + XbaI (at the 3' end) restriction sites and subcloned into a pTrc 99a vector (Harvard Medical School, Boston, MA) at the NcoI and XbaI sites. The molecular mass of the protein was 15,404 Da and the specific absorbance at 280 nm (calculated by DNAman program) was 2.62. Soluble protein was expressed upon induction with Isopropyl β-D-1-thiogalactopyranoside IPTG (Sigma). The protein was purified by successive extraction, ammonium sulfate precipitation, dialysis and lyophilization in the presence of $NaHCO_3$ at an approximate protein-to-salt ratio of 1:1. Its concentration was calculated according to specific absorbance at 280 nm. The protein solubilized easily in DDW. Its purity was determined by SDS-PAGE in the presence of a reducing agent and by analytical gel filtration on a Superdex 75 column developed in the presence of 25 mM Tris-HCl + 300 mM NaCl, pH 8. The purity, as determined by both methods, was >95% and the molecular mass under non-denaturing conditions indicated that the protein is a monomer, as verified in the following.

Fermentation

After preliminary experiments documented good expression of an ~15 kDa protein in the soluble fraction of *E. coli* DH5α cells after induction with 0.4 mM IPTG, large-scale fermentation of 2.5 l (5 x 500 ml, in 2.5-l flasks) was carried out. Once the cells reached an absorbance of 0.9 at 600 nm, IPTG was added to 0.4 mM and 8 h later, the cells were centrifuged and frozen.

Solubilization

The frozen pellets were suspended in 200 ml of 10 mM Tris pH 8 and 10 mM EDTA supplemented with phenylmethylsulfonyl fluoride (PMSF, Sigma) at a final concentration of 0.1 mg ml^{-1} and incubated on a stirrer for 2 h at 4°C. Then 2 µl of benzoaseR nuclease (Novagen Co. cat no. 70746-3, 25.0 units per ml) was added and the suspension was further incubated for 1 h at 4°C. Subsequently, the suspension was sonicated in two 100-ml portions for 6–7 min under a 50% cycle program and centrifuged for 20 min at 12,000 *g*. The supernatant was divided into ten 20-ml aliquots and frozen.

Ammonium sulfate precipitation

Frozen supernatant (60 ml) was thawed and 11.6 g of ammonium sulfate (0–30%, saturation) was added. This suspension was then incubated on a stirrer for about 15 min at 4°C and centrifuged for 20 min at 12,000 *g*. The supernatant (~70 ml) was saved and precipitated again with 29.4 g ammonium sulfate to reach 70% saturation, incubated on a stirrer for 15 min at 4°C and centrifuged for 20 min at 12,000 *g*. The supernatant was removed and the pellet was solubilized with 60 ml of 10 mM Tris pH 8 containing PMSF (final concentration of 0.1 mg ml^{-1}) and dialyzed at 4°C against 3 x 2 l of 10 mM Tris pH 8 containing PMSF (0.1 mg ml^{-1}).

Anion-exchange chromatography

A Q-sepharose column (25 ml bed volume) was pre-equilibrated with 10 mM Tris pH 8 and PMSF (0.1%). The dialyzed preparation was applied to the column at 4 ml min^{-1}. The flow-through fraction was collected and the column was washed with 160 ml of the same buffer. Then Oly was eluted with 50 mM NaCl in 10 mM Tris pH 8 and PMSF (0.1%). The eluate was collected in 40-ml aliquots

and every tube was tested for protein content by measuring absorbance at 280 nm, and every other tube for appearance of monomeric Oly by size-exclusion chromatography (SEC).

Preparative SEC column purification

The Q-sepharose column eluate was concentrated to 60 ml using an Amicon ultrafiltration unit equipped with a 10-kDa cut-off membrane and then applied in three consecutive 20-ml portions on a preparative Superdex 75 column (bed volume 300 ml) pre-equilibrated with 25 mM Tris-HCl, pH 8 containing 300 mM NaCl and developed at 4°C with at a flow rate of 2 ml min^{-1} (Supplementary Figure 1). After 1 h, 5-ml fractions were collected. Every tube was tested for protein content by measuring absorbance at 280 nm and every other tube for appearance of monomeric Oly by SEC and SDS-PAGE (Supplementary Figure 2). Tubes 8–14 were then pooled for final formulation. Note that this step removed some unknown material having an absorbance at 260 nm as the 280/260 ratio after SEC increased.

Formulation

The monomeric protein-containing pooled fractions were extensively dialyzed against Na-bicarbonate at a 1:1 (w/w) salt-to-protein ratio and lyophilized in 150- to 500-µg aliquots. The SEC and SDS-PAGE profiles of purified lyophilized recombinant Oly (rOly) are presented in Supplementary Figure 3. SEC analysis indicated that the purified rOly exists as a monomer under non-denaturing conditions. This was confirmed by the appearance of a single band of ~15 kDa by SDS-PAGE. SDS-PAGE carried out without the reducing agent showed identical results (not shown).

Preparation of the specific rOly polyclonal antibody

Two rabbits were immunized four times to obtain polyclonal antibody using 2 mg rOly, and producing 80 ml serum after 3 months. The antibody was purified using an affinity column construction after coupling the peptides to BSA and sepharose beads and purifying antigen-specific antibodies from 20–60 ml of rabbit sera. We obtained 2 mg purified antibody from the sera.

SUPPLEMENTARY FIGURES

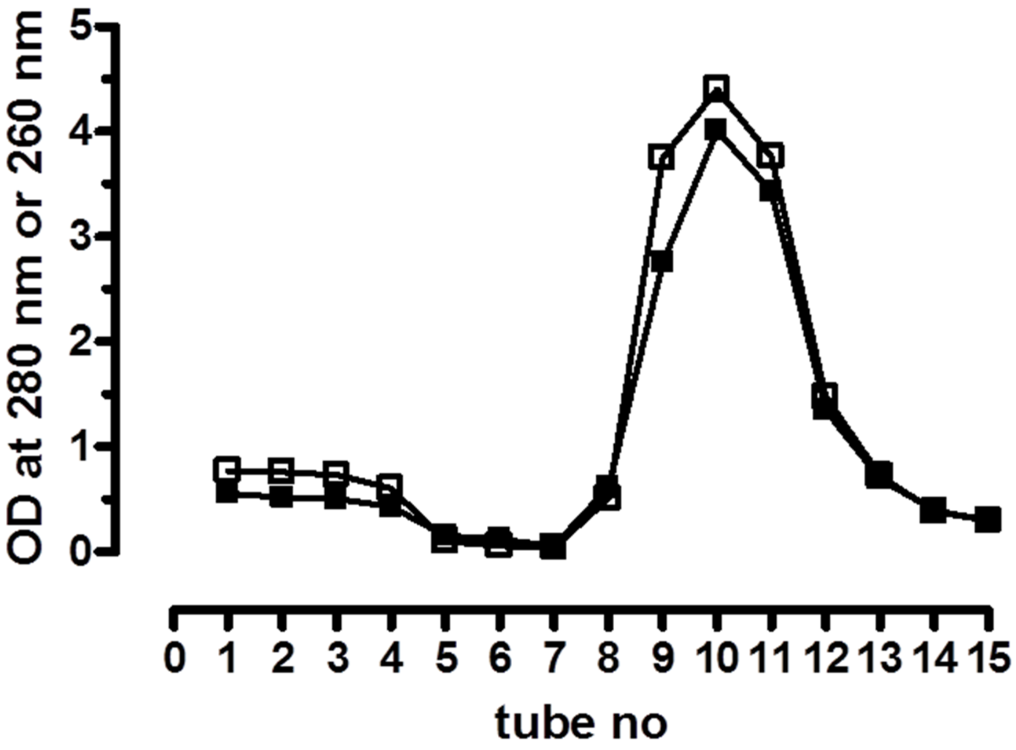

Supplementary Figure S1: Anion-exchange chromatography on Q-sepharose pre-equilibrated with 10 mM Tris pH 8 and PMSF (0.1%) of dialyzed ostreolysis after precipitation with ammonium sulfate. Tubes 1–3: flow-through fraction; tubes 4–7: washout fraction; tubes 8–20, 50 mM NaCl eluate. Each tube contained 40 ml of eluate and the separation was carried out at 4°C. Tubes 8–13 were pooled and used for the next purification step. Full squares – 280 nm, empty squares 260 nm.

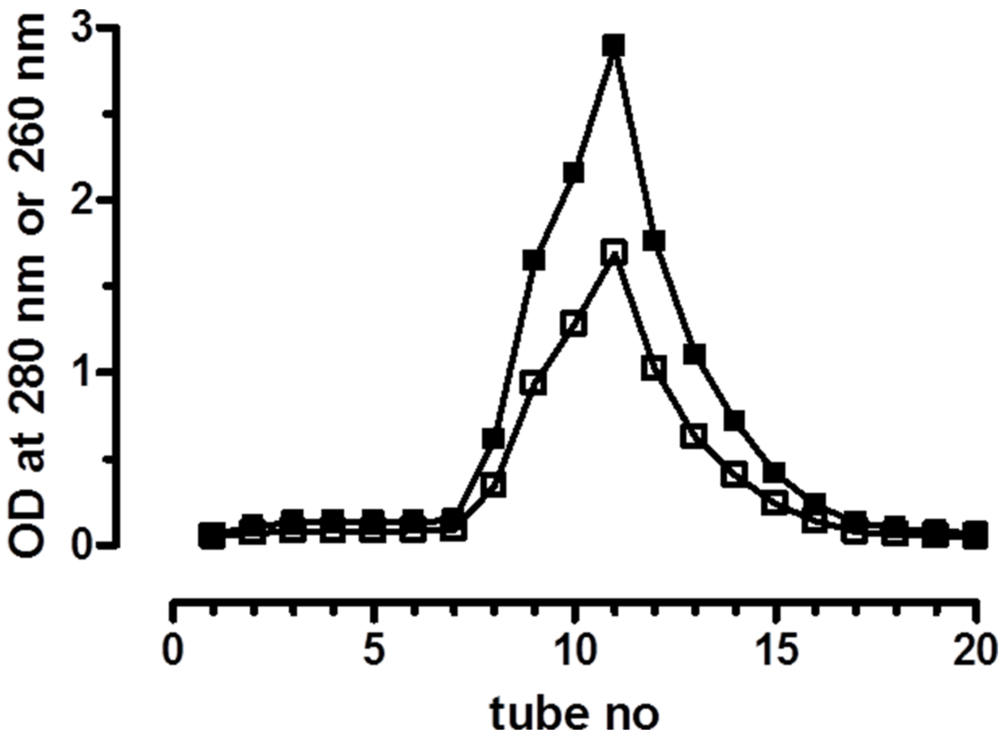

Supplementary Figure S2: Size-exclusion chromatography on preparative Superdex 200 column pre-equilibrated with 25 mM Tris pH 8 containing 300 mM NaCl. The separation was carried out at 4°C and 5-ml fractions were collected. Tubes 8–14 were pooled. Full squares – 280 nm, empty squares - 260 nm.

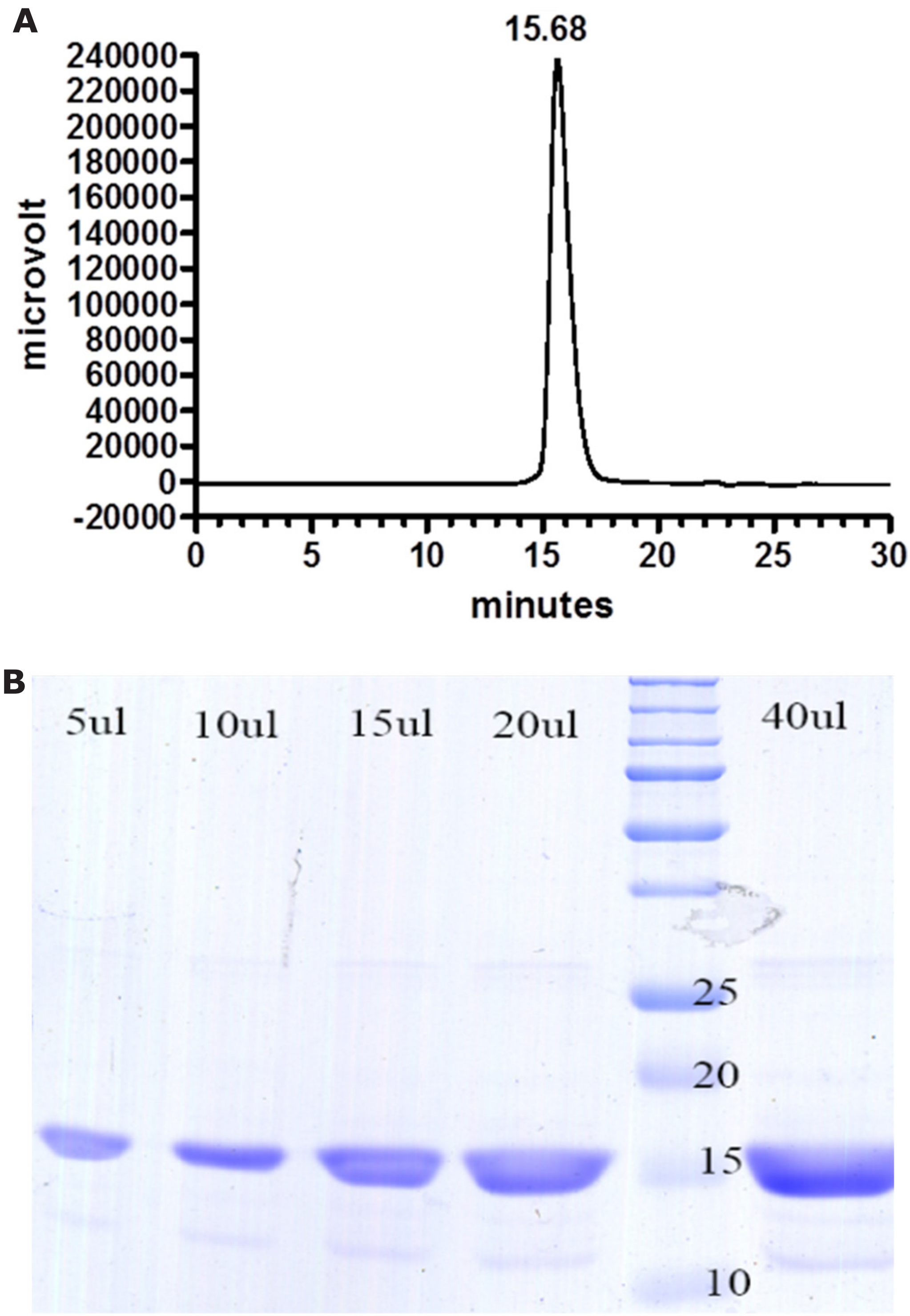

Supplementary Figure S3: A. Size-exclusion chromatography of the purified ostreolysin on analytical Superdex 75 column pre-equilibrated with 25 mM Tris pH 8 containing 300 mM NaCl. **B.** SDS PAGE (15%) in the presence of reducing agent. The 5–40 µl applied to each lane correspond, respectively, to 1–8 µg protein. The retention time of 15.68 min corresponds to a molecular mass of 16 kDa, as calibrated with BSA (66 kDa), human growth hormone (21.5 kDa) and leptin (16 kDa).
